# Population-Level Trends in Attention-Deficit/Hyperactivity Disorder Medication Prescribing

**DOI:** 10.1001/jamanetworkopen.2025.48532

**Published:** 2025-12-11

**Authors:** Daniel T. Myran, Rachael MacDonald-Spracklin, Giovanna Busa, Robert Talarico, Yaron Finkelstein

**Affiliations:** 1 Department of Family and Community Medicine, North York General Hospital, Toronto, Ontario, Canada; 2 Department of Family Medicine, University of Ottawa, Ottawa, Ontario, Canada; 3 Bruyère Health Research Institute, Ottawa, Ontario, Canada; 4 Ottawa Hospital Research Institute, Ottawa, Ontario, Canada; 5 ICES uOttawa, Ottawa Hospital Research Institute, Ottawa, Ontario, Canada; 6 School of Epidemiology and Public Health, Faculty of Medicine, University of Ottawa, Ottawa, Ontario, Canada; 7 Division of Emergency Medicine, Department of Paediatrics, The Hospital for Sick Children, Toronto, Ontario, Canada; 8 Department of Pharmacology and Toxicology, University of Toronto, Toronto, Ontario, Canada; 9 Division of Clinical Pharmacology and Toxicology, Department of Paediatrics, The Hospital for Sick Children, Toronto, Ontario, Canada

## Abstract

This cross-sectional study describes changes in prescription rates for attention-deficit/hyperactivity (ADHD) disorder medication in Ontario from 2015 to 2023.

## Introduction

Attention-deficit/hyperactivity disorder (ADHD) is a common neurodevelopmental disorder, affecting 1.6% to 5.0% of the population globally.^1^ Stimulant treatment for ADHD (eg, amphetamines) is associated with improved health and social outcomes.^2^ However, increasing stimulant prescribing in high-income countries has raised concerns about potential overdiagnosis, misuse, and adverse effects. We investigated whether population-level changes occurred in stimulant prescribing overall and across age and sex groups.

## Methods

This population-based, repeated cross-sectional study captured all residents of Ontario and all stimulant prescriptions dispensed in Ontario through the Narcotics Monitoring System. This study was approved by ICES, and informed consent was waived because the study used deidentified health information. The study followed the STROBE reporting guideline.

Health administrative datasets were linked using encoded identifiers and analyzed at ICES. We identified all stimulant prescriptions dispensed to all individuals aged 5 to 105 years between 2015 and 2023. We compared annual trends in rates of individuals with an incident prescription (no stimulant prescription in the previous 3 years) and individuals with a prevalent past-year prescription. Negative binomial regression models calculated average annual percentage change with 95% CIs, overall and stratified by age and sex. We tested for differences in trends over time using an interaction term between year and 2020. Analyses were conducted with SAS Enterprise Guide, version 7.1 (SAS Institute Inc). eMethods 1-3 in Supplement 1 provide included databases, prescription stimulants, and modeling details.

## Results

The study included 15 084 455 individuals (mean [SD] age, 42.3 [22.9] years; 50.7% female, 49.3% male), with 3.9% having 1 or more stimulant prescription. Overall annual incident stimulant prescriptions increased by 157.2%, from 275.2 per 100 000 individuals in 2015 to 708.0 in 2023. Incident stimulant prescription rates accelerated starting in 2020 (interaction *P* < .001), by 29.2% (95% CI, 24.9%-33.6%) per year between 2020 and 2023 compared with 7.4% (95% CI, 4.9%-9.9%) per year between 2015 and 2019 (Figure). Between 2015 and 2023, increases were higher in women than men. Largest increases were observed in age groups 25 to 44 (females, 421.3%; males, 219.7%), 18 to 24 (females, 368.7%; males 127.6%), and 45 to 64 (females, 188.3%; males, 119.0%) years, whereas the smallest increases occurred in persons aged 65 years or older (females, 17.9%; males, 2.3%) and 5 to 9 years (females, 49.1%; males, 32.7%).

**Figure.**
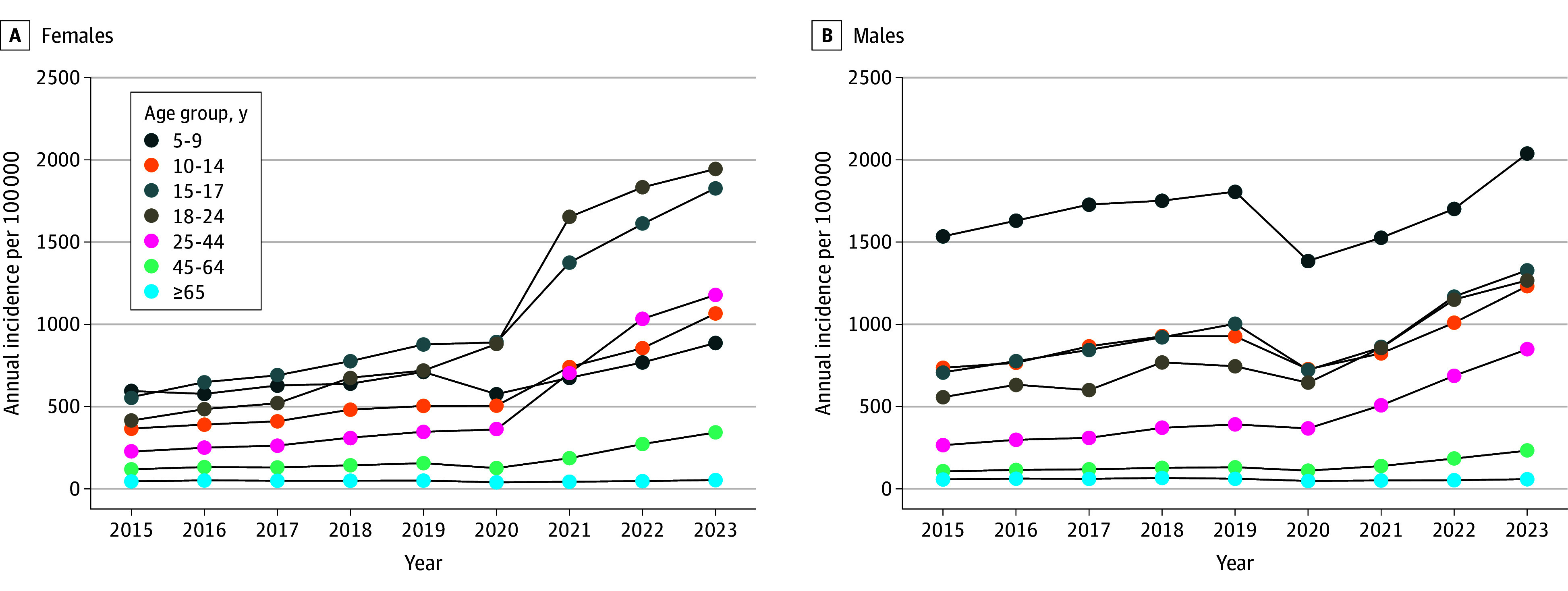
Trends in the Annual Incidence of Dispensed Stimulant Prescriptions in Ontario, Canada, Between 2015 and 2023, by Age and Sex

In 2023, prevalence of past-year stimulant prescriptions was 2.6% for the whole population, with the highest level at 7.8% in males aged 10 to 14 years and 6.7% in women aged 18 to 24 years. Greater increases in stimulant prescribing in females resulted in higher prevalence than in males aged 18 to 24 (6.7% vs 5.2%), 25 to 44 (3.8% vs 3.1%), and 45 to 64 (1.4% vs 1.1%) years in 2023 (Table).

**Table.** Trends in Incidence and Prevalence Rates of Dispensed Stimulant Prescriptions Between 2015 and 2023, by Age and Sex

Group	2023 Population, No.	2015		2023		Change in incidence over time			
		No. (rate per 100 000) with incident prescription	No. (%) with past year prescription	No. (rate per 100 000) with incident prescription	No. (%) with past year prescription	Relative change in rates, 2023 vs 2015, %		AAPC (95 CI), %	
								2015 to 2019	2020 to 2023
Overall	13 468 738	34 130 (275.2)	150 710 (1.2)	95 353 (708.0)	351 879 (2.6)	157.2	7.4 (4.9 to 9.9)		29.2 (24.9 to 33.6)
**Male age group, y**									
5-9	379 825	5689 (1534.9)	17 233 (4.6)	7739 (2037.5)	20 330 (5.4)	32.7	4.0 (2.9 to 5.2)		13.6 (11.8 to 15.4)
10-14	395 529	2729 (735.6)	23 313 (6.3)	4874 (1232.3)	30 824 (7.8)	67.5	6.7 (5.1 to 8.4)		19.7 (17.2 to 22.3)
15-17	241 367	1668 (708.2)	10 972 (4.7)	3207 (1328.6)	15 744 (6.5)	87.6	9.1 (7.1 to 11.0)		23.7 (20.7 to 26.7)
18-24	562 239	3373 (566.9)	14 816 (2.4)	7125 (1267.2)	29 058 (5.2)	127.6	8.1 (4.4 to 12.0)		26.2 (20.0 to 32.6)
25-44	1 865 701	4404 (265.3)	18 110 (1.1)	15 826 (848.3)	58 646 (3.1)	219.7	10.5 (8.5 to 12.5)		32.4 (29.2 to 35.7)
45-64	1 888 919	1984 (105.9)	9792 (0.5)	4382 (232.0)	20 318 (1.1)	119.0	5.4 (4.0 to 6.8)		28.6 (26.5 to 30.6)
≥65	1 276 917	550 (57.2)	1842 (0.2)	747 (58.5)	3493 (0.3)	2.3	1.9 (−0.6 to 4.4)		6.6 (3.0 to 10.4)
**Female age group, y**									
5-9	360 604	2092 (594.4)	5493 (1.6)	3196 (886.3)	7577 (2.1)	49.1	4.8 (3.4 to 6.2)		15.3 (13.2 to 17.4)
10-14	375 596	1289 (366.8)	7738 (2.2)	4002 (1065.5)	14 929 (4.0)	191.0	8.9 (5.9 to 11.8)		26.6 (22.1 to 31.2)
15-17	228 782	1246 (557.6)	5043 (2.3)	4182 (1827.9)	12 596 (5.5)	227.8	11.5 (7.4 to 15.7)		25.7 (19.4 to 32.3)
18-24	532 146	2389 (414.9)	9247 (1.6)	10 348 (1944.6)	35 506 (6.7)	368.7	15.4 (8.0 to 23.4)		27.5 (15.5 to 40.8)
25-44	1 882 417	3898 (226.1)	14 737 (0.9)	22 184 (1178.5)	71 953 (3.8)	421.3	11.3 (5.2 to 17.9)		48.1 (36.1 to 61.2)
45-64	1 966 375	2291 (118.8)	10 502 (0.5)	6784 (342.5)	27 087 (1.4)	188.3	6.4 (4.0 to 8.9)		40.0 (35.6 to 44.5)
≥65	1 512 312	528 (45.3)	1872 (0.2)	807 (53.4)	3818 (0.3)	17.9	1.5 (−1.0 to 4.1)		10.4 (6.7 to 14.3)

Abbreviation: AAPC, average annual percentage change.

## Discussion

This population-based, repeated cross-sectional study identified increases in stimulant prescribing, accelerating in 2020, particularly among females and individuals aged 18 to 64 years. Findings are consistent with prior research in commercially insured US populations^3^; however, our population-wide study reveals a broader and more accelerated increase in stimulant prescribing.

Several explanations may account for the increase and acceleration in stimulant prescribing. Findings may reflect true increases in individuals with ADHD or related symptoms due to social and environmental changes. For example, greater digital media use and online work and recreation, which are associated with development of ADHD symptoms,^4^ increased during COVID-19. Findings may also reflect improved identification of undiagnosed ADHD or misdiagnosis or overdiagnosis. Increases in online content on adult ADHD diagnosis and emergence of private virtual clinics offering ADHD assessments starting in 2020 may contribute to both improved identification and overdiagnosis.^5^

Study limitations include lack of data on prescription appropriateness. The rapid increase in stimulant use, particularly in adults, underscores the need for enhanced clinical training and evidence-based guidance in adult ADHD diagnosis. Prescription stimulant use is associated with increased risk of certain adverse outcomes regardless of indication,^6^ and growing prescription stimulant use may have important population-level health implications.

## Notes

Supplement 1. **eMethods 1.** Data Sources **eMethods 2.** List of Prescription Stimulants **eMethods 3.** Outcomes and Analytical Methods

Supplement 2.
Data Sharing Statement
