# Supplementary material for: Population-Level Trends in Attention-Deficit/Hyperactivity Disorder Medication Prescribing
Source: JAMA Netw Open. 2025 Dec 11;8(12):e2548532. doi: 10.1001/jamanetworkopen.2025.48532 (PMC12699356; doi:10.1001/jamanetworkopen.2025.48532)
Supplement: Supplement 1. — eMethods 1. Data Sources eMethods 2. List of Prescription Stimulants eMethods 3. Outcomes and Analytical Methods [file jamanetwopen-e2548532-s001.pdf]

Supplementary Online Content

Myran DT, MacDonald-Spracklin R, Busa G, Talarico R, Finkelstein Y. Population-level trends in attention-deficit/hyperactivity disorder medication prescribing. *JAMA Netw Open*. 2025;8(12):e2548532. doi:10.1001/jamanetworkopen.2025.48532

eMethods 1. Data Sources

eMethods 2. List of Prescription Stimulants

eMethods 3. Outcomes and Analytical Methods

This supplementary material has been provided by the authors to give readers additional information about their work.

© 2025 Myran DT et al. *JAMA Network Open*.

eMethods 1. Data Sources

We obtained study data from de-identified and linked health administrative databases housed at ICES (formerly Institute of Clinical Evaluative Sciences). ICES is an independent, non-profit research institute funded by an annual grant from the Ontario Ministry of Health and Long-Term Care. As a prescribed entity under Ontario's privacy legislation, ICES is authorized to collect and use health care data for the purposes of health system analysis, evaluation and decision support. Secure access to these data is governed by policies and procedures that are approved by the Information and Privacy Commissioner of Ontario.

The dataset from this study is held securely in coded form at ICES. While legal data sharing agreements between ICES and data providers (e.g., healthcare organizations and government) prohibit ICES from making the dataset publicly available, access may be granted to those who meet pre-specified criteria for confidential access, available at www.ices.on.ca/DAS (email: das@ices.on.ca). The full dataset creation plan and underlying analytic code are available from the authors upon request, appreciating that the computer programs may rely upon coding templates or macros that are unique to ICES and are, therefore, either inaccessible or may require modification.

The following datasets were linked using unique encoded identifiers and were analyzed at ICES:

- Stimulant prescriptions were obtained from the Narcotics Monitoring System (NMS) database, which contains comprehensive records of prescriptions for stimulants dispensed from pharmacies in Ontario, regardless of payer.
- The population at risk was obtained from the registered Persons Database (RPDB), which includes the total number of persons at risk each month and individuals' age and sex, along with their eligibility for Ontario's public insurance.

© 2025 Myran DT et al. *JAMA Network Open*.

eMethods 2. List of Prescription Stimulants

Drug Identification Number (DIN)	Brand Name	Generic Name
02439239	Act Amphetamine XR	Dextroamphetamine mixed salts and Amphetamine
02439247	Act Amphetamine XR	Dextroamphetamine mixed salts and Amphetamine
02439255	Act Amphetamine XR	Dextroamphetamine mixed salts and Amphetamine
02439263	Act Amphetamine XR	Dextroamphetamine mixed salts and Amphetamine
02439271	Act Amphetamine XR	Dextroamphetamine mixed salts and Amphetamine
02439298	Act Amphetamine XR	Dextroamphetamine mixed salts and Amphetamine
02448319	Act Dextroamphetamine SR	Dextroamphetamine Sulfate
02448327	Act Dextroamphetamine SR	Dextroamphetamine Sulfate
02441934	Act Methylphenidate ER	Methylphenidate Hydrochloride
02441942	Act Methylphenidate ER	Methylphenidate Hydrochloride
02441950	Act Methylphenidate ER	Methylphenidate Hydrochloride
02441969	Act Methylphenidate ER	Methylphenidate Hydrochloride
02248808	Adderall XR	Dextroamphetamine mixed salts and Amphetamine
02248809	Adderall XR	Dextroamphetamine mixed salts and Amphetamine
02248810	Adderall XR	Dextroamphetamine mixed salts and Amphetamine
02248811	Adderall XR	Dextroamphetamine mixed salts and Amphetamine
02248812	Adderall XR	Dextroamphetamine mixed salts and Amphetamine
02248813	Adderall XR	Dextroamphetamine mixed salts and Amphetamine
02445492	Apo-Amphetamine XR	Dextroamphetamine mixed salts and Amphetamine
02445506	Apo-Amphetamine XR	Dextroamphetamine mixed salts and Amphetamine
02445514	Apo-Amphetamine XR	Dextroamphetamine mixed salts and Amphetamine
02445522	Apo-Amphetamine XR	Dextroamphetamine mixed salts and Amphetamine
02445530	Apo-Amphetamine XR	Dextroamphetamine mixed salts and Amphetamine
02445549	Apo-Amphetamine XR	Dextroamphetamine mixed salts and Amphetamine
02443236	Apo-Dextroamphetamine	Dextroamphetamine Sulfate
02546647	Apo-Lisdexamfetamine Capsules	Lisdexamfetamine Dimesylate
02546655	Apo-Lisdexamfetamine Capsules	Lisdexamfetamine Dimesylate
02546663	Apo-Lisdexamfetamine Capsules	Lisdexamfetamine Dimesylate
02546671	Apo-Lisdexamfetamine Capsules	Lisdexamfetamine Dimesylate
02546698	Apo-Lisdexamfetamine Capsules	Lisdexamfetamine Dimesylate
02546701	Apo-Lisdexamfetamine Capsules	Lisdexamfetamine Dimesylate
02546728	Apo-Lisdexamfetamine Capsules	Lisdexamfetamine Dimesylate
02249324	Apo-Methylphenidate	Methylphenidate Hydrochloride
02249332	Apo-Methylphenidate	Methylphenidate Hydrochloride

© 2025 Myran DT et al. *JAMA Network Open*.

02273950	Apo-Methylphenidate	Methylphenidate Hydrochloride
02330377	Apo-Methylphenidate ER	Methylphenidate Hydrochloride
02452731	Apo-Methylphenidate ER	Methylphenidate Hydrochloride
02452758	Apo-Methylphenidate ER	Methylphenidate Hydrochloride
02452766	Apo-Methylphenidate ER	Methylphenidate Hydrochloride
02266687	Apo-Methylphenidate SR	Methylphenidate Hydrochloride
02277131	Biphentin	Methylphenidate Hydrochloride
02277158	Biphentin	Methylphenidate Hydrochloride
02277166	Biphentin	Methylphenidate Hydrochloride
02277174	Biphentin	Methylphenidate Hydrochloride
02277182	Biphentin	Methylphenidate Hydrochloride
02277190	Biphentin	Methylphenidate Hydrochloride
02277204	Biphentin	Methylphenidate Hydrochloride
02277212	Biphentin	Methylphenidate Hydrochloride
02247732	Concerta	Methylphenidate Hydrochloride
02247733	Concerta	Methylphenidate Hydrochloride
02247734	Concerta	Methylphenidate Hydrochloride
02250241	Concerta	Methylphenidate Hydrochloride
01924516	Dexedrine	Dextroamphetamine Sulfate
01924559	Dexedrine Spansules	Dextroamphetamine Sulfate
01924567	Dexedrine Spansules	Dextroamphetamine Sulfate
02470292	Foquest	Methylphenidate Hydrochloride
02470306	Foquest	Methylphenidate Hydrochloride
02470314	Foquest	Methylphenidate Hydrochloride
02470322	Foquest	Methylphenidate Hydrochloride
02470330	Foquest	Methylphenidate Hydrochloride
02470349	Foquest	Methylphenidate Hydrochloride
02470357	Foquest	Methylphenidate Hydrochloride
02326221	Methylphenidate	Methylphenidate Hydrochloride
02326248	Methylphenidate	Methylphenidate Hydrochloride
02326256	Methylphenidate	Methylphenidate Hydrochloride
02315068	Novo-Methylphenidate ER-C	Methylphenidate Hydrochloride
02315076	Novo-Methylphenidate ER-C	Methylphenidate Hydrochloride
02315084	Novo-Methylphenidate ER-C	Methylphenidate Hydrochloride
02315092	Novo-Methylphenidate ER-C	Methylphenidate Hydrochloride
02126486	Phl-Methylphenidate	Methylphenidate Hydrochloride
02126494	Phl-Methylphenidate	Methylphenidate Hydrochloride

© 2025 Myran DT et al. *JAMA Network Open*.

02246991	Phl-Methylphenidate	Methylphenidate Hydrochloride
02440369	PMS-Amphetamines XR	Dextroamphetamine mixed salts and Amphetamine
02440377	PMS-Amphetamines XR	Dextroamphetamine mixed salts and Amphetamine
02440385	PMS-Amphetamines XR	Dextroamphetamine mixed salts and Amphetamine
02440393	PMS-Amphetamines XR	Dextroamphetamine mixed salts and Amphetamine
02440407	PMS-Amphetamines XR	Dextroamphetamine mixed salts and Amphetamine
02440415	PMS-Amphetamines XR	Dextroamphetamine mixed salts and Amphetamine
00584991	PMS-Methylphenidate	Methylphenidate Hydrochloride
00585009	PMS-Methylphenidate	Methylphenidate Hydrochloride
02234749	PMS-Methylphenidate	Methylphenidate Hydrochloride
02536943	PMS-Methylphenidate CR	Methylphenidate Hydrochloride
02536951	PMS-Methylphenidate CR	Methylphenidate Hydrochloride
02536978	PMS-Methylphenidate CR	Methylphenidate Hydrochloride
02536986	PMS-Methylphenidate CR	Methylphenidate Hydrochloride
02536994	PMS-Methylphenidate CR	Methylphenidate Hydrochloride
02537001	PMS-Methylphenidate CR	Methylphenidate Hydrochloride
02537028	PMS-Methylphenidate CR	Methylphenidate Hydrochloride
02537036	PMS-Methylphenidate CR	Methylphenidate Hydrochloride
02413728	PMS-Methylphenidate ER	Methylphenidate Hydrochloride
02413736	PMS-Methylphenidate ER	Methylphenidate Hydrochloride
02413744	PMS-Methylphenidate ER	Methylphenidate Hydrochloride
02413752	PMS-Methylphenidate ER	Methylphenidate Hydrochloride
02541602	Quillivant ER Chewable Tablets	Methylphenidate Hydrochloride
02541610	Quillivant ER Chewable Tablets	Methylphenidate Hydrochloride
02541629	Quillivant ER Chewable Tablets	Methylphenidate Hydrochloride
02541637	Quillivant ER Oral Suspension	Methylphenidate Hydrochloride
02230321	Ratio-Methylphenidate	Methylphenidate Hydrochloride
02230322	Ratio-Methylphenidate	Methylphenidate Hydrochloride
02247364	Ratio-Methylphenidate	Methylphenidate Hydrochloride
00005606	Ritalin	Methylphenidate Hydrochloride
00005614	Ritalin	Methylphenidate Hydrochloride
00632775	Ritalin SR	Methylphenidate Hydrochloride
02457288	Sandoz Amphetamine XR	Dextroamphetamine mixed salts and Amphetamine
02457296	Sandoz Amphetamine XR	Dextroamphetamine mixed salts and Amphetamine
02457318	Sandoz Amphetamine XR	Dextroamphetamine mixed salts and Amphetamine
02457326	Sandoz Amphetamine XR	Dextroamphetamine mixed salts and Amphetamine
02457334	Sandoz Amphetamine XR	Dextroamphetamine mixed salts and Amphetamine

© 2025 Myran DT et al. *JAMA Network Open*.

02457342	Sandoz Amphetamine XR	Dextroamphetamine mixed salts and Amphetamine
02546248	Sandoz Lisdexamfetamine Capsules	Lisdexamfetamine Dimesylate
02546256	Sandoz Lisdexamfetamine Capsules	Lisdexamfetamine Dimesylate
02546264	Sandoz Lisdexamfetamine Capsules	Lisdexamfetamine Dimesylate
02546272	Sandoz Lisdexamfetamine Capsules	Lisdexamfetamine Dimesylate
02546280	Sandoz Lisdexamfetamine Capsules	Lisdexamfetamine Dimesylate
02546299	Sandoz Lisdexamfetamine Capsules	Lisdexamfetamine Dimesylate
02546302	Sandoz Lisdexamfetamine Capsules	Lisdexamfetamine Dimesylate
02320312	Sandoz Methylphenidate SR	Methylphenidate Hydrochloride
02533340	Taro-Lisdexamfetamine Chewable Tablets	Lisdexamfetamine Dimesylate
02533359	Taro-Lisdexamfetamine Chewable Tablets	Lisdexamfetamine Dimesylate
02533367	Taro-Lisdexamfetamine Chewable Tablets	Lisdexamfetamine Dimesylate
02533375	Taro-Lisdexamfetamine Chewable Tablets	Lisdexamfetamine Dimesylate
02533383	Taro-Lisdexamfetamine Chewable Tablets	Lisdexamfetamine Dimesylate
02533391	Taro-Lisdexamfetamine Chewable Tablets	Lisdexamfetamine Dimesylate
02545861	Teva-Lisdexamfetamine	Lisdexamfetamine Dimesylate
02545888	Teva-Lisdexamfetamine	Lisdexamfetamine Dimesylate
02545896	Teva-Lisdexamfetamine	Lisdexamfetamine Dimesylate
02545918	Teva-Lisdexamfetamine	Lisdexamfetamine Dimesylate
02545926	Teva-Lisdexamfetamine	Lisdexamfetamine Dimesylate
02545934	Teva-Lisdexamfetamine	Lisdexamfetamine Dimesylate
02545942	Teva-Lisdexamfetamine	Lisdexamfetamine Dimesylate
02322951	Vyvanse	Lisdexamfetamine Dimesylate
02322978	Vyvanse	Lisdexamfetamine Dimesylate
02347156	Vyvanse	Lisdexamfetamine Dimesylate
02347164	Vyvanse	Lisdexamfetamine Dimesylate
02347172	Vyvanse	Lisdexamfetamine Dimesylate
02439603	Vyvanse	Lisdexamfetamine Dimesylate
02458071	Vyvanse	Lisdexamfetamine Dimesylate
02490226	Vyvanse	Lisdexamfetamine Dimesylate
02490234	Vyvanse	Lisdexamfetamine Dimesylate
02490242	Vyvanse	Lisdexamfetamine Dimesylate
02490250	Vyvanse	Lisdexamfetamine Dimesylate
02490269	Vyvanse	Lisdexamfetamine Dimesylate
02490277	Vyvanse	Lisdexamfetamine Dimesylate

© 2025 Myran DT et al. *JAMA Network Open*.

eMethods 3. Outcomes and Analytical Methods

Incident and Prevalent Prescriptions

Prescription data from the NMS was available starting in 2012. We began our analytic period in 2015 to ensure that all years had a complete three-year look-back data for identifying incident prescriptions. Prevalent Prescriptions were calculated as the proportion of eligible individuals in each calendar year who received one or more prescriptions for a stimulant.

Analytical Approach

We fit negative binomial regression models where the outcome was the total number of individuals each year in the age and sex stratum, with either an incident stimulant prescription or past year stimulant prescription as separate outcomes. We selected negative binomial models instead of Poisson models as there was evidence of overdispersion. Models were offset by the natural log of the Ontario population at-risk each year. The population at risk was identified on January 1st of each year.

We included year as a continuous integer variable in the model. Model coefficients were then exponentiated to obtain rate ratios and then transformed by subtracting 1 and multiplying by 100 to return an average annual percent change (AAPC) and 95% confidence intervals. To assess changes in trends over time, we included an interaction between year and a binary indicator for period (2015-2019=0, 2020-2023=1). We then computed the AAPC for both periods separately based on the regression coefficients of the product term and reported the p-value for the interaction between year and period.

Models were completed overall for the entire Ontario population and separately for each age and sex stratum (e.g., boys aged 5-9 years, girls aged 5-9 years). As the study used population-level data rather than samples, formal statistical comparison tests of differences in changes between groups based on age or sex stratum were not performed as the changes represent the observed change.

© 2025 Myran DT et al. *JAMA Network Open*.
