# Supplementary material for: Population-Level Trends in Attention-Deficit/Hyperactivity Disorder Medication Prescribing
Source: JAMA Netw Open. 2025 Dec 11;8(12):e2548532. doi: 10.1001/jamanetworkopen.2025.48532 (PMC12699356; doi:10.1001/jamanetworkopen.2025.48532)
Supplement: Supplement 2. — Data Sharing Statement [file jamanetwopen-e2548532-s002.pdf]

Data Sharing Statement

Myran. Population-Level Trends in Attention-Deficit/Hyperactivity Disorder Medication Prescribing. *JAMA Netw Open*. Published December 11, 2025. doi:10.1001/jamanetworkopen.2025.48532

Data
Data available: No
